# Peripheral artery disease (PAD) in primary care—educational experiences for PAD primary care in England—a mixed-method study

**DOI:** 10.1093/fampra/cmad048

**Published:** 2023-04-21

**Authors:** Bernadeta M Bridgwood, Rob D Sayers

**Affiliations:** Cardiovascular Sciences, University of Leicester, Leicester, United Kingdom; Cardiovascular Sciences, University of Leicester, Leicester, United Kingdom

**Keywords:** atherosclerosis, cardiovascular disorders, chronic disease, DVT, health promotion, hypertension, peripheral arterial disease, prevention, primary care

## Abstract

**Background:**

Peripheral artery disease (PAD), the pathophysiologic narrowing of arterial blood vessels of the lower leg due to atherosclerosis, is a highly prevalent disease, with sharp increases in prevalence with age. Primary care is ideally located to identify and manage PAD.

**Objectives:**

This study aims to identify the educational experiences, opinions, and confidence of primary care clinicians (PCCs) regarding PAD.

**Method:**

This mixed-method study was conducted within primary care in England. An online survey was completed with follow-on semistructured interviews, between January and September 2021, with PCCs, namely GPs, practice nurses, and allied professionals (survey *n* = 874, interviews *n* = 50).

**Results:**

PCCs report variation in PAD education received, where the content could not often be recalled. Patient-focussed experiential and self-directed learning, formed the largest method to gain PAD education. All PCCs recognized that they have an important role in recognizing PAD yet confidence in recognizing and diagnosing PAD was lacking. PCCs acknowledged that late or missed PAD diagnosis resulted in significant patient morbidity and mortality. Yet many did not recognize PAD as a common disease.

**Conclusion:**

As “specialist–generalists” with finite resources, education provided to primary care needs to be applicable for the multimorbid patient presentations often seen, utilizing resources available in primary care, with consideration to the time constraints endured.

**Key messages**

- Clinicians acknowledge an important role in recognizing PAD.
- Confidence in recognition/diagnosis was lacking.
- Clinicians report variable PAD education received—content could not be recalled.
- PAD primary care education needs consideration to multimorbidity.
- Experiential and patient-focussed learning forms an important form of education.

## Background

Peripheral arterial disease (PAD) is an atherosclerotic process that leads to significant arterial narrowing or blockage supplying the lower limbs. It is common disease and is an independent risk factor for coronary and cerebral disease.^1^ PAD has an estimated worldwide prevalence of almost 10%, increasing to 20% in those over 70 years of age.^2,3^ However, many patients are minimally or asymptomatic, hence it is frequently under-recognized and is often only identified at advanced stages.^1,4^ PAD is associated with significant morbidity and mortality.^4^ Although incurable, early recognition with ensuing lifestyle and medical management can reduce life-changing sequela.^5^

Primary care has a significant role in primary/secondary prevention of chronic disease and is principally responsible for implementing the National Institute for Health and Care Excellence (NICE) guideline for lower-limb PAD in the United Kingdom.^6^ However, primary care clinicians (PCCs) awareness of the PAD diagnosis is relatively low.^2,3,7^ Consequential atherosclerosis risk factors, including lipid disorder and hypertension, though very prevalent in PAD patients are less intensively treated in comparison with patients with cardiovascular disease (CVD). Under-recognition and diagnosis of PAD in primary care practice reduces opportunity for effective secondary prevention.^6^ To support improved PAD management, PCCs need to be aware of and recognize PAD. The aim of this study was to identify PAD education provided for PCCs which has not previously been identified. This information was not available from any accessible sources including online medical school curriculum nor when individual universities were contacted. Therefore, it was aimed to gain this information directly from PCCs which provided opportunity to explore PCCs confidence and opinions of PAD.

## Methods

Primary care practices across England registered their interest and consent to participate through the National Institute of Health Research Primary Care clinical research network (January to September 2021).

### Online survey

The survey was hosted on JISC online and categorized into 5 sections: (i) participant information/consent, (ii) PCC educational experiences, (iii) PAD knowledge-based screening, (iv) PCC confidence with PAD, and (v) educational preferences. The online surveys were sent to the practice study lead who then disseminated the survey to their PCCs. Surveys were completed by participants remotely and independently. Results were tabulated and analysed within Statistical Package for Social Sciences (V28). Association of knowledge and diagnostic confidence was determined using Chi-square test. A copy of the survey can be found in Supplementary Information.

### Semistructured interviews

PCC’s received a participant information sheet prior to the interview and signed a consent form, including for digital recording. An interview guide included topics of prior education, recognition of PAD and educational preferences. This had previously been tested on 10 GPs for validity. One hundred and twelve PCCs registered interest via email and 50 were able to participate within the timeframe. None withdrew. Interviews were conducted via MSTeams between the lead author (BMB) and PCC participant for a maximum of 15 min, with voice-only recording to uphold participant confidentiality. In situ member checking occurred to minimize resource impact on PCCs. Researcher reflexive notes were made after each interview to detail initial thoughts.^8^ Each interview was transcribed verbatim and managed using NVivo (version 12). All were analysed using an inductive thematic analysis, where each theme was developed iteratively within data sessions with BMB and RDS with repeated re-reading, open coding, and mapping to reflect the content of the data. BMB and RDS have different clinical perspectives (primary care/vascular surgery, respectively), which aimed to reduce reflexivity of a single speciality perspective. Interpretive analysis allowed for the refining of the specifics of themes and thematic patterns, and a final coding frame was developed.^9^

## Results

### Surveys

Surveys were completed by 874 PCCs from 189 practices (55% urban; 45% rural, practice response rate 84%)—505 general practitioners and 41 GP trainees (doctors), 260 nurses, 38 advanced practitioners (APs), and 30 clinical practitioners (CPs) (including health care assistants, home-visiting parademics, and podiatry). Participants came from Yorkshire and Humber; the North East, West Midlands, East of England, London, and the South East and West. All patient-facing practitioners were included to capture the diverse roles of PCCs. Results from the survey are compiled in Table 1.

**Table 1.** Survey results of 874 PCCs tabulating their experiences of PAD education, knowledge of the National PAD guidance, confidence in diagnosis and identification of PAD and educational resources utilized (2021).

	Participant groups	Doctors	Nurse	APs	CPs
PCC participant information	*Time since qualification*				
	Mean	17 years	21 years	16 years	Not given
	Median	14 years	22 years	14 years	
	Range	3 months to 47 years	<1 to 44 years	8 months to 46 years	
	*Self-identified role*—*ranked by PCC*				
	Diagnosis	1	4	1	3
	Admission prevention	5	6	4	4
	Medication review	3	5	3	—
	Wound management	6	2	6	2
	Prescribing medications to reduce CVD risk	2	3	5	—
	Nonmedication risk factor management	4	1	2	1
PAD educational experience	Undergraduate level	24%	11%	21%	4%
	Postgraduate level e.g. GP/nurse training	23%	20%	21%	15%
	Experiential learning	22%	26%	23%	23%
	Personal reading	17%	19%	19%	27%
	Others including through CPD/professional education memberships	12.5%	11%	7%	12%
	No training	1.5%	13%	9%	19%
Knowledge screening	Correct description identified for intermittent claudication (%)	87%	61%	76%	69%
	NICE guideline adherent management	88%	68%	72%	52%
PCC confidence with PAD	Ability to recognize symptomatic PAD—very confident	10%	3%	0%	6%
	Confident	48%	13%	29%	35%
	Somewhat confident	32%	52%	56%	12%
	Not confident	10%	33%	15%	47%
	Any PAD training/education was significantly associated with increased confidence of recognizing PAD (*χ*^2^ = 596.67, *P* < 0.001) Length of time in service did confer increased confidence/perceived knowledge (*χ*^2^ = 530.48, *P* < 0.001)				
	Ability to recognize presence/absence of pulses—very confident	20%/11%	28%/20%	21%/9%	24%/24%
	Confident	59%/48%	43%/33%	44%/29%	35%/35%
	Somewhat confident	20%/34%	24%/39%	26%/47%	41%/41%
	Not confident	2%/7%	5%/8%	9%/15%	0%/0%
	PAD training/education was significantly associated with increased confidence of identifying the presence of pulses (*χ*^2^ = 240.08, *P* < 0.001) and absence of pulses (*χ*^2^ = 628.45, *P* < 0.001)				
Resources used	Existing knowledge 35% NICE guideline/CK summaries 27% GP notebook 15%	Google 10% Other available sources 12% None 1%			

### Interviews

A total of 40 GPs (time since qualifying 3/40 within 5 years, 12/40 with 10 years and the remainder over 20 years), 8 practice nurses (2 qualified within 2 years, remainder over 20 years since qualification), and 2 allied professionals (qualified within 3 years) participated. Data saturation occurred at participant 42, with additional information collected to substantiate the findings. Quotations are presented with participant identifier codes in the format of job role followed by participant number (Dr—doctor; N—nurse; A—allied professional).

#### Theme 1—PAD education

##### Subtheme 1—undergraduate

Less than half of PCCs had received PAD education during undergraduate training (22/50) which had usually taken the form of either a lecture or small group work. PCCs described this as 1 (21/22) or 2 sessions, usually incorporated into a cardiovascular or diabetes teaching module. All described that they could recall minimal theoretical information. Where a practical element was involved, ABPI was taught was remembered (12/22).

“Basic coverage at medical school…in the cardiovascular block” Dr6

“I remember vaguely being taught how to feel pedal pulses and it being very difficult” Dr30

“I still remember a practical session on ABPI and I do ABPI on my patients now when I suspect PAD…” Dr25

##### Subtheme 2—postgraduate

10/50 PCCs had never received any PAD teaching which had extended into postgraduate training. Only 1 PCC remembered any PAD education during GP training, none in nursing or allied professional training. Over 75% of PCCs described self-directed learning through other methods including personal reading and external education events. This was instigated by the PCC recognizing an educational need, for example following a patient interaction.

“There is no formal training once you qualify” Dr27

“In GP training, did training on dopplers” Dr8

“We discussed clinical scenarios in local teaching” Dr32

#### Theme 2—engaging with general education within primary care

All PCCs engaged in educational activities whether this was internal or external interactions. PCCs require education that can be utilized and applied to primary care; for example, information of focussed examinations designed for a 10-min consultation, first line management and referral pathways.

“…(an educator should be) knowledgeable, up-to-date, has practical experience with general practice as well as secondary care, because sometimes you have these specialists, they only deal one in their own little world, and they don’t look at the whole bigger picture.” Dr47

“If led by secondary care consultant they often focus on secondary care speciality and their own pet procedures aspects which is not relevant for primary care.” Dr31

Primary care consultations are not often focussed on a single disease. Indeed, they consider multimorbidity, psychological and social aspects and require education to reflect this.

“Often you get teaching sessions put at you from a single diseases perspective. There are so many ‘things’ you need to know as a GP. So sometimes combining things could be helpful” Dr25

“We’re generalists aren’t we? So we need to be aware of everything, particularly, you know, diseases like peripheral vascular disease which are not isolated. I mean I don’t come across many people with peripheral vascular disease who don’t have other comorbidities…” Dr10

Most of the PCCs described some form of, and greatly valued, experiential learning. This was either a direct interaction with patients with PAD or indirectly by learning from a colleagues’ direct patient interactions. Hence, utilizing patient cases as an educational tool provides grounding of the information. When synergies are recognized between patients, PCCs discussed disease recognition, transferability and application of their learning.

“I’d say actually my biggest learning experiences have come from in practice and patient stories” Dr20

“my greatest knowledge…(has come)… from seeing patients, experience of examining them, assessing them and referring them on and being told what ive done right and what ive done wrong” Dr24

The COVID-19 pandemic has changed PCCs perspectives on how to interact with education. Converting to virtual learning offered in a variety of formats including webinars and podcasts, increased accessibility and attendance despite long working hours and child-care requirements. PCCs described new found motivation to participate, especially if education is delivered free of charge. However, networking opportunities are strongly valued and there still remained a preference for in-person interaction rather than virtual.

“A lot of the webinars are becoming free and online education and I think that is a catalyst for a lot of people to enjoying the learning process” Dr7

“After I just got home from a long day at work, I’m much more motivated to join those (virtual) sorts of meetings than I would be to drive halfway across Derbyshire for a 2 hour meeting…” Dr10

“…there is great value in talking with colleagues, especially the ‘corridor’ conversations with other who face the am challenges—you can’t do that online…” N37

Being an educator also provides motivation to keep up to date and engage with education. Many participants were involved in under or postgraduate education and as such described bidirectional learning with their trainees, with other trainers or colleagues.

“I’m a GP trainer. I meet regularly with other trainers in our work and trainers workshop that is probably the type of meeting I find most useful. We have daily clinical meetings at our practice where everyone talk can talk about cases and referrals and stuff” Dr41

“I think that’s the way I keep up to date really, because… the trainees ask you questions and…then you might go away and research it together or they tell you things that they found out” Dr50

#### Theme 3—clinician attitudes towards PAD

##### Subtheme 1—PAD as a disease entity

PCCs (38/50) referred to PAD as “not a common disease,” particularly when comparing to other diseases and “yesterdays” disease. PCCs stated that they would consider “more common” diseases such as osteoarthritis before they would consider PAD. Several PCCs suggested that if there were more funding available to promote PAD, such as that undertaken for heart attacks and strokes, then it may be more often considered. Other motivators for considering diseases in primary care include measured indicators under the Quality and Outcomes Framework (QoF) which denote points and associated funding. Currently, 2 QoF points are awarded for establishing and maintaining a register of patients with PAD. This is compared with 80 points available for diabetes, including monitoring and diagnosis. Moreover, there is no lead clinician representing PAD from a local or national perspective which there are for many other diseases.

“You know the squeaky wheel gets the grease sort of thing, so whatever has been promoted to us as being important it’s what we know about” N5

“I think, like most people I would walk through the more common differentials first before that (PAD) comes to mind” Dr30

“Maybe I think in GP land because we don’t get any Qof point (for PAD), you don’t get any money toward PAD and why we think about diseases like diabetes and COPD, where you do get payment towards it” Dr2

##### Subtheme 2—recognizing PAD

PCCs repeatedly discussed uncertainty towards recognizing PAD when a patient does not present with a “classical presentation of PAD,” that of calf pain when walking and improving on rest. As stated above, clinicians consider other diagnoses first.

“(I would consider PAD) if they come with that sort of very classic claudication history which most patients don’t tend to because they haven’t read the same textbooks as we have, so they’re not going to describe it to you the way you would” A39

“I certainly don’t have much experience in it…particularly ulceration side of things is managed sort of nurse managed isn’t it rather than GP managed, so that ends comes with those tools with difficult ones, which I feel I’m not very well prepared to manage” Dr10

PCCs reflected on the PAD education they had received and the expectations of their job. For example, more than half of the nurse practitioners interviewed, who were regularly performing diabetic foot checks, had minimal or no training in conducting a vascular examination. Although many discussed experiential learning, there were examples of missed PAD diagnoses.

“As I say ive never had any training on it, even from a student nursing up to masters degree is never really anything that’s ever been mentioned and you are expect to do PAD checks. I think it is being missed.” N1

“I had a patient recently with an ulcer who was directed to the nurse who said that she could feel the pulses and he wasn’t diabetic and I’m thinking this is a bit strange. So then I called him in and you couldn’t feel any pulses and it was the critically ischemic foot.” Dr31

PCCs overwhelmingly acknowledged that there was an issue with missed or late presentations of PAD which have resulted in missed opportunities for primary or secondary prevention. Indeed, patients present with end stage complications of PAD. PCCs partially attributed this to not being considered, a lack of confidence when assessing pulses or considered within a silos of being an ulcer. Ulcers are commonly managed in primary care, however PCCs suggest these are not always considered to being linked to another disease process.

“We see loads of patients with ulcers it’s just trying to make sure that they’re being managed, really effectively, and that we’re not missing the diagnosis, which I fear is happening a lot in some patients they just have months and months of dressings and no one actually checks that they haven’t got peripheral artery disease earlier on” N36

“I’ve seen a cluster of patients who have presented reasonably late with their symptoms, just ‘cause they’ve been purely not picked up in primary care earlier” Dr6

“PAD. It does get missed, doesn’t it?” Dr8

Despite the above, all clinicians identified that PAD is a condition that any patient-facing clinician should be able to recognize and that there is much that primary care can offer in terms of primary and secondary prevention and management.

“We are absolutely best placed to give lifestyle advice and identifying which of our patients are more likely to have peripheral arterial disease and then secondary care” A36

“I mean, when I first started doing this, a lot of it was all sent straight to the hospital for assessment, whereas there’s a lot more you can do in primary Care now before you send them off” Dr3

PCCs also considered the role of educating patients to be aware of PAD as a condition, especially in those at risk, for example in those with CVD or diabetes and those who smoke. This aims to empower patients and enhances autonomy in managing their own health.

“…it might just not just be education on the part of the clinicians it might be education on the path of the patients as well” Dr4

“I think the more people that get educated around, anything to do with patient health I think is good, but especially PAD” N12

## Discussion

This study was undertaken to identify the PAD educational experiences of practising primary care practitioners including their confidence in its recognition and exploration of attitudes. Primary care is considered by PCCs to be well positioned to recognize and manage chronic disease such as PAD. However, a lack of knowledge in the public through to clinicians for PAD is widely reported.^7,10,11^ PCCs report variable experience of PAD education during all stages of training with no reported formal education by 1.5% of doctors up to 19% by CPs. Any PAD education was described within multidisease teaching, such as with diabetes or CVD, which is very appropriate to this condition.^12^ Consequentially PCCs lacked knowledge in considering PAD as a diagnosis and lacked confidence when diagnosing and managing risk factors associated with PAD.

Practical sessions were remembered with more detail, where ABPI was often remembered years later. Here, learners interact experientially with the information and practical information was retained for longer, particularly if comparative theoretical knowledge requires retention for shorter periods e.g. for a forthcoming examination.^13^ Outside of formal education, PAD knowledge was gained primarily through experiential learning and by PCC-sought CPD including personal reading. Here, PCCs report motivation to gain knowledge prompted by personal or colleague clinical experiences. Such learning engages sociocultural learning theories (SCTs), tying learning to patient context, facilitating learning through participation and active engagement in the activities of the community.^14,15^

Despite increasing prevalence and being regarded as a common condition in published literature,^16^ PCCs describe PAD as “not common” considering other diseases such as osteoarthritis as a diagnosis before PAD. Moreover, atypical PAD presentations also led to diagnostic uncertainty. Confidence in recognizing symptoms of PAD correlated with this where between 40% and 85% of each clinical group self-rating as either somewhat confident or not confident at all. This is indirectly conceded within a wider forum as it boasts no regional or national leads, little regard in QoF indicators and little mention in public media. Current perspectives of UK PAD screening within primary care demonstrate heterogeneous opinions as although QoF motivates GP practices to have a register of patients with PAD, there is currently no incentive to screen asymptomatic patients.^17^ Moreover, PAD screening is not currently recommended by the National Screening Centre.^18^ However, PCCs acknowledge that they should be able to recognize patients at risk, or symptoms of, PAD.^19^ PCCs identified significant roles in PAD diagnosis, yet confidence of recognizing symptoms varied from 0% to 56% across the clinical groups. Significantly, the majority of nurses who identified a major role in wound management, which is often when PAD may be recognized, had no PAD training at undergraduate level or while training to be a nurse and 33% were not confident at recognizing symptomatic PAD. Increased time in service correlated with increased confidence and perceived PAD knowledge, again exemplifying SCTs.

UK primary care offers annual health screening undertaken for a range of diseases including hypertension, diabetes, and CVD, providing ample opportunity for PAD screening within at risk populations.^20–22^ In particular, if patients are specifically asked about claudication symptoms, this could improve PAD identification.^17^ Automation within software templates during patient interactions, such as annual reviews, could provide an opportunity to probe for PAD. However, PAD requires a higher profile both by patients, for instance, through simple posters in the waiting room, through to more complex media channels and by clinicians, for example through QoF indicators and available education.

In this study, there was an appetite for improving patient PAD awareness through patient education. In another study, a lack of patient education, little follow-up, and a general lack of PCC attention paid to patients PAD diagnosis led patients to conclude that their condition did not warrant the same attention as other diseases such as diabetes.^23^

The literature consistently encourages improvement of clinician knowledge through education.^19^ Much clinical education has been based on a secondary care curriculum. However, education for PCCs needs to be primary care focussed with due consideration to how a clinician can interact and utilize the information within a short consultation, while considering multimorbidity and incorporating patient-focussed education. Many PCCs believed that improved primary care focussed PAD education aimed at all clinicians could address the issue of under-recognition of PAD. However, improved knowledge does not consistency translate to improved patient care.^24^ PCCs utilize a range of resources to support PAD management yet existing knowledge remains the most utilized, followed by NICE guidance. The PAD UK-wide guideline provides evidence-based direction for PCCs who are generalists^20^ and an important tool for learning.^6^ When testing PCCs knowledge of this guideline, the average knowledge score was 80% in PAD diagnosing clinicians. In comparison, 68% of nurse and 52% of CPs had guideline adherent knowledge. The next most utilized resource was google, which when queried in the interviews, PCCs were then directed to any of the aforementioned resources with NICE guidance the most popular onward resource.

### Strengths and limitations

A limitation of this study was that clinicians participating may have a particular interest in PAD. Including the large number of PCCs past the saturation point allowed themes to be developed iteratively and confirmed the themes. As current PAD education could not be obtained, we relied on PCCs memory, where education had often been undertaken some time previous. However, this gave a realistic view of PCCs working memory/retained information and attitudes towards PAD. Furthermore, the view of PAD educators and relevant stakeholders was not obtained. These may have shed light on the current climate for PAD education. However, the study did reinforce the need for education tailored specifically for primary care. A further limitation was that the personal teaching experiences of the study group may have influenced the interpretation of the results. However, in situ member checking and interpretation by the study group who came from different specialities aimed to minimize bias in the interpretation.

### Implication for research and practice

This study has given insight into the preferences of how to address education within primary care. There was little published evidence identified to specially address this. Although an introduction to PAD within undergraduate education would be useful, postgraduate education, particularly learning from patient cases or experiential learning, including a practical element and utilization of SCTs would compliment primary care. Interprofessional education may facilitate interactive patient case-based discussion and PAD education rooted within a multimorbidity context would reflect patient interactions. Ideally, there should be overlap with consultation skills focussed for a concise yet thorough consultation. Moreover, practical skills that require no resources or those available in any primary care setting e.g. primary care focussed pulse examinations without the use of equipment such as dopplers. Providing online resources may increase PAD education engagement although in-person events are valued for the networking opportunities. Finally, integration of claudication screening questions with patient education within annual patient reviews could lead to increased recognition of PAD at an earlier stage.

## Supplementary Material

cmad048_suppl_Supplementary_Material_S1

cmad048_suppl_Supplementary_Material_S2

cmad048_suppl_Supplementary_Material_S3

## Data Availability

The data underlying this article will be shared on reasonable request to the corresponding author.
