# Supplementary material for: Peripheral artery disease (PAD) in primary care—educational experiences for PAD primary care in England—a mixed-method study
Source: Fam Pract. 2023 Apr 21;40(5-6):820–6. doi: 10.1093/fampra/cmad048 (PMC10745240; doi:10.1093/fampra/cmad048)
Supplement: cmad048_suppl_Supplementary_Material_S1 [file cmad048_suppl_supplementary_material_s1.docx]

**Supplementary Information – Topic/Prompt guide for interview**

Note: the following observation prompt guides are flexible tools which are open to revision if new areas of interest arise during the process of data collection. In order to adapt to any logistical factors (e.g. the subject and duration of observation) the points below are for guidance only.

In the each interview, the interviewer will remind the participant of the project aims and check that they fully understand the participant information sheet, and will then take them through the informed consent process. Audio-recording of the interview will then begin. Researcher observations and thoughts will be captured by the researcher making field notes.

Member checking will be undertaken to uphold validity of initial results analysis and to avoid additional impact on clinician time.

**INTERVIEWS WITH CLINICIANS**

Many thanks for agreeing to speak to me today. I am part of a larger research team that is interested in peripheral artery disease. I am currently looking into the educational opportunities that primary healthcare professionals have been exposed to, regarding peripheral arterial disease and how their opinions of PAD.

- Prompt guide
  - Demographic data or confirm information
    - Could you tell me your job role?
    - Where is your practice based?
    - Is it a rural/urban practice?
    - Could you briefly describe your patient demographics?
  - Could you tell me about any training or education you had about peripheral artery disease?

| If yes -   - What did you find helpful? - What was not helpful? - How could this have been improved? - Has the training been helpful for managing a patient with PAD? - How do you think educational opportunities will change after the COVID-19 pandemic? | If no –   - Could you describe whether training would be useful for your job role? - What might it look like? - How do you think educational opportunities will change after the COVID-19 pandemic? |
| --- | --- |

- - Can you describe an educational experience which was really useful?
    - Why was it?
  - How confident would you feel about recognising ‘symptomatic’ PAD which is claudication, critical limb ischemia? E.g. do you use information to make a possible diagnosis? E.g. from history alone or examination (mainly pulses) or Doppler assessments. Would you consider PAD to be a CVD risk factor?
- How confident do you feel when managing a patient with PAD?
  - What affects your confidence?
  - When would you consider referring a patient to vascular surgery?
- Do you think that PAD training is necessary? What do you think is achievable post COVID-19?

If you wish to receive a copy of the study results, please email [bb176@leicester.ac.uk](mailto:bb176@leicester.ac.uk).
