# Supplementary material for: Peripheral artery disease (PAD) in primary care—educational experiences for PAD primary care in England—a mixed-method study
Source: Fam Pract. 2023 Apr 21;40(5-6):820–6. doi: 10.1093/fampra/cmad048 (PMC10745240; doi:10.1093/fampra/cmad048)
Supplement: cmad048_suppl_Supplementary_Material_S3 [file cmad048_suppl_supplementary_material_s3.docx]

**This study aims to explore the educational experience of UK primary care health practitioners for peripheral arterial disease (PAD), including identification of factors that shape preferred learning styles and assess health professionals opinions of PAD recognition and management.**

Thank you for considering this survey. The aim of this survey is to help us understand your experience of learning opportunities for PAD, your opinions on PAD management and continued professional development. **The survey should only take up to 10 minutes to complete**. **You will not be asked for your name, email address or any other contact details.**

Peripheral arterial disease (PAD) is a term used to describe the impairment of blood flow to the extremities usually as a result of ather​osclerotic occlusive disease. The main symptom of PAD is intermittent claudication (leg pain while walking), however as the disease progresses, PAD can cause critical limb ischemia (ulceration and gangrene) and lead to a requirement for amputation. Despite healthcare professionals within primary care often being the first to identify and manage PAD, evidence shows self-reported knowledge and understanding of the condition to be lacking.

1. a) Your Role Which best describes your professional role?

Nurse

Nurse in training

GP

GP registrar

Other (please state below)

b) If you are a GP or practice nurse, how many years has it been since you qualified?

2.What responsibilities do you have for the care of people with peripheral artery disease? Tick all that apply.

Diagnosis

Prescribing medications to reduce cardiovascular risk

Admission prevention

Wound management

No responsibility

Management of patient risk factors (non-medication) e.g. providing smoking cessation advice

Medication review

Other (please state below)

3.Have you received any education/training regarding PAD? Tick all that apply

Never

Personal reading

During GP/nurse training

Within an external education/training opportunity

Continued professional development within the practice

During primary/undergraduate medical/nursing qualification

Online education/training opportunity

Can not remember any training

Other (please state below)

4. Which of the following can be associated with intermittent claudication? Tick all that apply

Pain which occurs during rest

Pain located in the calf area

Pain that occurs during effort

Pain located in the buttocks area

Pain which improves with exertion

Pain that improves on rest

5. How confident would you feel about recognising 'symptomatic' PAD (e.g. intermittant claudication, critical limb ischaemia) in a patient?

Very confident

Confident

Somewhat confident

Not confident

Would not be able to recognise

6. Which of the following do you use to support screening and diagnosis of symptomatic PAD? Tick all that apply

Risk factors

Symptoms

Pulse examinations

Edinburgh claudication questionnaire

Co-morbidities

Ankle-brachial pressure index (ABPI)

Doppler

Other (please state below)

7. Does your practice have access to ABPI assessment? Tick all that apply

Yes in the practice

Yes through community services

Yes through secondary care referral

No access

Other (please state below)

8. How confident would you feel about identifying the PRESENCE of pulses in the feet of a patient?

Very confident

Confident

Somewhat confident

Not very confident

Would not be able to recognise

9. How confident would you feel about identifying the ABSENCE of pulses in the feet of a patient?

Very confident

Confident

Somewhat confident

Not very confident

Would not be able to recognise

10. In your opinion, which healthcare professional would be most effective at identifying 'symptomatic' PAD and why?

11. Do you use any resources when managing a patient with peripheral arterial disease? Tick all that apply

None

Existing knowledge acquired from education/training on PAD

NICE guidance/Clinical Knowledge Summaries (CKS) summary

GP notebook

Monthly Index of Medical Specialities (MIMS)

Google search

Guidelines in practice

Journal articles

Other (please state below)

12. What management options do you consider important for PAD? Tick all that apply

Exercise

Flu vaccination

Smoking cessation

Statin therapy

Diabetes management

Weight management

Aspirin as monotherapy antiplatelet

Blood pressure management

Report to DVLA (car-driver)

Clopidogrel as monotherapy antiplatelet

Referral to secondary care

Other (please state below)

13. When choosing to participate in continued professional development, what factors do you take into consideration? Tick all that apply

The topic is important for my job

The topic is interesting to me

Teaching is delivered face to face (with consideration to social distancing)

The teaching is free

I will receive CPD points

There is a short distance to travel to teaching

The subject is taught by medical consultants or consultant surgeons

There is a practical aspect to the teaching

Teaching is delivered in the evening

Teaching is delivered at the weekend

The subject is taught by GP experts

Teaching is delivered online

Satisfies learning needs identified following a patient consultation/contact

Teaching is delivered in the day

Teaching is delivered via an app

The subject is taught by specialist nurses

Other (please state below)

14. Which of the following teaching methods would you want to be used following the COVID-19 pandemic? Tick all that apply

Webinars

Practice level teaching

E-learning

Remote group teaching e.g. via zoom/Microsoft teams

In person (face-to-face) one to one education with an external teacher

Teaching delivered through mobile phone apps

Artificial intelligence teaching

Removal of a ‘teacher’

Group teaching (face to face)

Remote one to one education with an external teacher

Multiple-practice teaching

Other (please state below)

15. If educational resources were available for PAD, would this be of interest to you?

Yes

No
